# Research Progress of Ba(Zn_1/3_Nb_2/3_)O_3_ Microwave Dielectric Ceramics: A Review

**DOI:** 10.3390/ma16010423

**Published:** 2023-01-02

**Authors:** Sen Peng, Yu Zhang, Tulin Yi

**Affiliations:** 1 Provincial Key Laboratory of Informational Service for Rural Area of Southwestern Hunan, Shaoyang University, Shaoyang 422000, China; 2 School of Physics and Electronics, Central South University, Changsha 410083, China; 3 Department of Basics, Air Force Early Warning Academy, Wuhan 430019, China

**Keywords:** Ba(Zn_1/3_Nb_2/3_)O_3_ ceramics, preparation method, performance modification, microwave dielectric properties

## Abstract

Ba(Zn_1/3_Nb_2/3_)O_3_ (BZN) microwave dielectric ceramics have attracted great attention due to their high-quality factor (*Q*), near-zero temperature coefficient of resonant frequency (τ*_f_*), and suitable dielectric constant (ε_r_), making them promising materials for application in microwave devices. Due to their superior dielectric properties, composite perovskite ceramics are widely used in the field of microwave communication, base stations, navigation, radar, etc. This article summarized the latest research progress of BZN ceramics and discusses the main preparation methods and performance modifications. Furthermore, the problems faced by BZN ceramics and solutions to improve their performance, as well as their potential applications, are analyzed. This article provides a reference for the design and preparation of BZN ceramics.

## 1. Introduction

Microwave dielectric ceramics are a new type of functional ceramic developed in the past 40 years, and a key basic material for manufacturing microwave components. Therefore, they are highly valued, and increasingly so, by many countries, especially those with developed electronic industries [1]. In recent years, these materials has been widely used in the manufacture of dielectric filters and resonators in portable mobile phones, car phones, television satellite receivers, military radars and other aspects, playing a huge role in modern communications [2]. Based on the dielectric constant, microwave dielectric ceramics can be divided into the following three types: low permittivity ceramics (ε_r_ < 30) [3], moderate permittivity ceramics (30 < ε_r_ < 70) [4], and high permittivity ceramics (ε_r_ > 70) [5]. Among them, moderate permittivity ceramics are mainly used as dielectric resonators in military radars and communication systems in the 4~8 GHz frequency band, requiring high *Q* value and near-zero τ*_f_* value. Nevertheless, with the miniaturization, high frequency, high reliability and low cost of electronic products, the requirements for the performance of microwave dielectric ceramics have become higher and higher. The high permittivity ceramics reduce the size of devices. The high *Q* value is conducive to improving the selectivity of the operating frequency. The near-zero τ*_f_* value is beneficial in improving the temperature stability of devices [6].

In recent years, ceramic compounds of Ba(X_l/3_Y_2/3_)O_3_ (X = Mg, Zn, Co, Ni; Y = Nb, Ta) with composite perovskite structures, as shown in Table 1, have attracted more extensive attention. The medium material based on this compound can better conform to the requirements of larger ε_r_, high *Q* × *f* value and near-zero τ*_f_*, and can be used for the preparation of filters and resonators. In the current research system, the most typical representative of a microwave dielectric ceramic with a low dielectric constant is Ba(Zn_1/3_Ta_2/3_)O_3_ (BZT), and its dielectric performances are shown below: ε_r_ = 28, *Q* × *f* = 168,000 GHz. τ*_f_* = 0.5 ppm/°C [7]. Although the performances of BZT ceramic are outstanding, raw material Ta_2_O_5_ with high purity is very expensive, its sintering temperature is too high (usually higher than 1600 °C), and the holding time too long (~20 h) [8]. Therefore, it is not easy to manufacture, and the high cost ultimately limits its large-scale application. However, Nb_2_O_5_ is a kind of compound with a low price and extensive sources, which can also be used to replace Ta_2_O_5_ to produce dielectric ceramics with good performance under appropriate conditions [9]. Therefore, Ba(Zn_1/3_Nb_2/3_)O_3_ (BZN) ceramics have great advantages in the application of microwave devices. At present, the research hotspot of BZN ceramics mainly focuses on the optimization of microwave dielectric properties and the influencing factors of dielectric properties, especially the *Q* × *f* value and τ*_f_* value [10]. The influencing factors of microwave dielectric properties mainly include microstructure, density, conductivity, stoichiometry, porosity, etc. [11,12]. In addition, environmental factors, such as temperature, also have a particular influence on microwave dielectric properties [13,14]. Moreover, the quality factor, permittivity, and density of BZN ceramics can be improved by doping NiO [15]. However, it is easy to form additional phase BaO in the sintering process, leading to an inconspicuous effect on improvement of the quality factor *Q* [15]. Therefore, some researchers reduce the sintering temperature by annealing [16,17], or adding sintering co-solvents, such as MnCO_3_ and BaZrO_3_ [18], achieving superior microwave dielectric performance: ε_r_ = 43.6, tan*δ* = 0.6 × 10^−4^, τ*_f_* = −8 ppm/°C, the densification sintering temperature is reduced to 1240 °C, and the relative density reaches 95.18%.

In this paper, the dielectric properties of BZN ceramics over recent years are reviewed, the influencing factors are analyzed in detail, and the barium zinc niobate ceramic system is systematically introduced in order to provide some help for the study of the dielectric properties of barium zinc niobate microwave ceramics.

## 2. Structure and Properties of Ba(Zn_1/3_Nb_2/3_)O_3_

BZN ceramics possess excellent dielectric properties: ε_r_ = 41, *Q* × *f* = 86,925 GHz (*f* = 9.5 GHz), τ*_f_* = +31 ppm/°C, and the lattice constant a = 0.4094 nm. BZN ceramics are compatible with perovskite structures (including hexagonal and cubic structures). In an octahedron, the radius of Zn^2+^ and Nb^5+^ is small, and the larger radius Ba^2+^ and O^2-^ ions combine to form a dense cubic stacking structure. Since the charges and radii of Zn^2+^ and Nb^5+^ are quite different, they tend to be regularly arranged in the <111> direction of the crystal, that is, along the <111> direction of the crystal, in a molar ratio of 1:2, which changes the crystal structure from irregular cubic to a regular hexagonal ordered superstructure [25]. The ordered structure of BZN ceramics leads to low dielectric loss. Although BZN ceramics possess good dielectric properties, if they are applied to communication equipment under current communication conditions, the *Q* value needs to be improved and the τ*_f_* value should be near-zero, so as to make it work normally and stably under various temperatures. In addition, how to lower the sintering temperature is also one of the research directions for sintered ceramics.

## 3. Approaches to Fabricate BZN Ceramics

In this paper, the key technologies for preparing BZN ceramics were studied, such as the solid-state ceramic route, coprecipitation, citrate gel, spray pyrolysis, and inverse microemulsion.

### 3.1. Solid-State Ceramic Route

At present, microwave dielectric ceramics are mainly prepared by the solid-state ceramic route. The primary process of this method includes mixing the powder according to the chemical proportion, ball milling, drying and calcining, then re-milling, drying, granulating, tablet pressing again, and, finally, calcining. The solid-state ceramic route has the advantages of simple operation and being low cost, thus it is suitable for mass production.

C.L. Huang et al. [26] synthesized 0.95Ba(Zn_1/3_Nb_2/3_)O_3_-0.05BaZrO_3_ by a conventional solid-state method in order to study its microwave dielectric properties. The results revealed that the samples sintered at 1300–1500 °C did not exhibit an ordered structure, due to the formation of liquid phases. When increasing the sintering temperature, the grain growth rate was different, so that the density of the samples decreased. The dielectric constant displayed the same changing trend as density, and the peak value was obvious when the sintering temperature was 1450 °C. At 1500 °C, the ceramic sample possessed the lowest temperature coefficient, which was ascribed to the formation of redundant liquid phases. The above analysis showed that the 0.95Ba(Zn_1/3_Nb_2/3_)O_3_-0.05BaZrO_3_ sample, sintered at 1450 °C for 2 h, could obtain the best performance: *Q* × *f* = 96,000 GHz (*f* = 7 GHz), ε_r_ = 42, τ*_f_* ≈ +25 ppm/°C.

Generally speaking, for ceramics, annealing treatment could promote grain growth and improve crystallinity. In addition, proper ion arrangement could eliminate defects and residual stress resistance, which was useful to improve the *Q* value of sintered ceramics [27].

F. Azough et al. [28] studied the effects of the annealing process on the ordering structure of BZN ceramics. It was found that samples cooled at 600 °C/h exhibited a cubic structure, and the XRD patterns of the samples cooled at 60 °C/h or 5.0 °C/h all displayed a 1:2 ordered hexagonal structure. With decrease in the cooling rate, the *Q* × *f* value increased gradually, which might be due to the 1:2 ordered structure.

### 3.2. Coprecipitation Method

Coprecipitation means that the solution contains two or more cations, and there is a homogeneous phase in the solution. It is an important method for preparing composite oxide ultrafine powder containing two or more metal elements. The advantages of the coprecipitation method are shown below. For one thing, nanopowder materials with uniform chemical composition can be acquired using different chemical reactions in solution. Other additional advantages include easy preparation and uniform distribution of grains [29,30].

A. Mergen [31] prepared BZN ceramics using the coprecipitation method. It was found that nano BZN ceramics powder was synthesized by the coprecipitation method, and the BZN phase could be achieved at 400 °C. Pure BZN ceramics could be obtained at a low sintering temperature of 800 °C. The microstructure of coprecipitation powder showed that the prepared grains were spherical in shape, and the spherical diameter was in the range of 90–120 nm. BZN ceramics sintered at 1250 °C and showed the following performance: high density (relative density was about 95%), dielectric constant ε_r_ = 39, dielectric loss tan*δ* = 7.4 × 10^−4^, and grain size less than 1 um.

### 3.3. Citrate Gel Method

The citrate gel method dissolves the nitrate of each metal in ethylenediamine solution of citric acid. The solution is heated to 90–120 °C for 1–2 h, and then cooled to room temperature, so as to form a uniform gel. The gel is degraded for 2 h at 500 °C to remove impurities in the sample. After this, the steps are similar to those for solid phase reaction, such as calcination and sintering, and, thus, a superconducting powder is finally obtained [32,33]. This method is a chemical method, which is suitable for the production of nanopowder. It has never been used to produce BZN ceramics before. Compared with the mixed oxide route, the citrate gel method can form BZN at a lower temperature.

D. Sert [34] reported that nanoscale BZN microwave dielectric ceramic powders could be prepared by the citrate gel method. Compared with the traditional mixed oxide route, BZN ceramics with perovskite structures were formed at lower temperatures. Scanning electron microscopy and transmission electron microscopy showed that although the BZN powder was agglomerated to a certain extent, the grain size was between 70–110 nm, spherical and evenly distributed.

### 3.4. Spray Pyrolysis Method

Spray pyrolysis is a method of injecting metal salt solution into high-temperature gas in the form of a mist so that the solvent evaporates rapidly, the metal salt decomposes, and then the solid phase is precipitated by supersaturation, thus obtaining the nanometer powder directly. Another way is to spray the solution at high temperature, dry it in the atmosphere, and then heat-treat it to form powder. The grain size formed by this method has a great relationship with the spraying process parameters. However, this method needs to be carried out under vacuum and high temperature. It has high requirements for equipment and operation, but i easily to produces dust with small grain size and good dispersion [35,36].

M.H. Liang et al. [37] observed two reactions during the heating of BZN powder prepared by spray pyrolysis. The first reaction was the decomposition of residual hydrocarbons at 300 °C–400 °C, and the second reaction was the evaporation of ZnO at 840 °C. Cubic perovskite BZN (*Q* × *f* = 180,000 GHz at 13.5 GHz) could be successfully obtained from the synthesized powders by spray pyrolysis. Therefore, it was very important to strictly control the calcination and sintering process, which could prevent the loss of ZnO by vaporization. According to observation, the best condition for calcining spray pyrolysis powder was at 800 °C/4 h, which was just below the temperature of ZnO vaporization.

### 3.5. Inverse Microemulsion Method

The inverse microemulsion method is a new material preparation method developed in recent years. It is a material preparation method obtained by looking for one or more microemulsion methods to synthesize grains with different sizes and shapes, obtaining the required properties of materials [38].

Y.C. Lee et al. [39] reported that BZN powders could be prepared by the inverse microemulsion method. The prepared powder had amorphous clusters of about 100 nm, which could be directly transformed into a pure perovskite phase without forming a mesophase or secondary phase. When the sintering temperature reached 1400 °C, the prepared BZN ceramics showed microwave dielectric properties: the *Q* × *f* value reached 15,500 GHz, and the relative density reached 94%.

## 4. Performance Modification of BZN Ceramics

### 4.1. A-Site Ion Doping

The ion radii difference between Ca^2+^ and Ba^2+^ is more than 15%. For A-site ions, calcium ions are small, so calcium ions are easily cause vibration in the space of an A-site, and the vibration of calcium ions also leads to the distortion of the lattice from the original cubic structure to a hexagonal structure, resulting in the abnormality of the dielectric properties of (barium, calcium) system [40].

D. Liu et al. [40] studied (Ca_1−*x*_Ba*_x_*)(Zn_1/3_Nb_2/3_)O_3_ ceramics and they found that when (1-*x*) increased from 0.1 to 0.3, the permittivity decreased, which was due to the emergence of a Ca-rich second phase. The appearance of a second phase also reduced the τ*_f_* value, and the structure changed from an ordered structure to disorder, leading to a decrease of *Q* × *f* value. When Ca(Zn_1/3_Nb_2/3_)O_3_ was indexed to the main phase, the *Q* × *f* value gradually increased and was higher than that of (Ba_1−*x*_Ca*_x_*)(Zn_1/3_Nb_2/3_)O_3_ [41]. The microwave dielectric properties and unit cell volume for Ca_1−*x*_Ba*_x_*(Zn_1/3_Nb_2/3_)O_3_ ceramics are shown in Table 2.

Y.C. Liou [42] successfully prepared perovskite (Ba*_x_*Sr_1−*x*_)(Zn_1/3_Nb_2/3_)O_3_ (BSZN, *x* = 0.3, 0.5 and 0.7) ceramics through a reaction-sintering process. The results revealed that the second phases of ZnNb_2_O_6_ and (Cu_2_Zn)Nb_2_O_8_ were formed at the grain boundaries of the perovskite matrix as BSZN samples sintered at 1400–1500 °C for 2 h. Moreover, the content of the second phases in BSZN samples increased with increasing Sr^2+^ content. The densification sintering temperature of BSZN ceramics increased with increasing Sr^2+^ content. For example, Ba_0.7_Sr_0.3_(Zn_1/3_Nb_2/3_)O_3_ ceramics were sintered at 1400 °C to achieve a maximum density of 5.94 g/cm^3^. Ba_0.3_Sr_0.7_(Zn_1/3_Nb_2/3_)O_3_ ceramics were sintered at 1430–1500 °C to obtain a maximum density of 6.19 cm^3^. The temperature difference reached 30–100 °C. The results indicated that the reaction-sintering process not only improved the density of ceramic samples, but was also more effective than the traditional oxide process [43]. By observing scanning electron microscope (SEM) pictures, it could be found that with the increase of Sr^2+^ content, the microstructure of the samples became more and more compact. In addition, the samples with lower Sr^2+^ content were more likely to form secondary phases when sintered at the same temperature. The grain size was correlated with the sintering temperature and Ba^2+^ content, which was because the driving force for perovskite grain growth increased at higher temperatures [44]. This driving force grew with increasing Ba^2+^ content in BSZN ceramics.

A.F. Qasrawi et al. [45] prepared Ba_1−*x*_La*_x_*(Zn_1/3_Nb_2/3_)O_3_ by adding La^2+^ into BZN using traditional solid-state reaction technology to adjust the dielectric properties of BZN. It was found that Ba_5_Nb_4_O_15_ was obvious when *x* = 0.05, and Ba_3_LaNb_3_O_12_ was formed with *x* = 0.10. When *x* = 0.10 and 0.20, the mass fraction of Ba_3_LaNb_3_O_12_ was 7.6% and 60.47%, respectively, which meant that the dissolution of La^2+^ in BZN reached the limit and new substances were forming. The density and relative density of Ba_1−*x*_La*_x_*(Zn_1/3_Nb_2/3_)O_3_ increased with increasing La^2+^ content. Microcrystals could inhibit spontaneous polarization, and the permittivity increased with an increase of grain size. When *x* = 0.10, the conductivity of BZN ceramics increased from 1.62 to 9.24.

X. Wang et al. [46] prepared Ca_1−*x*_Ba*_x_*(Zn_1/3_Nb_2/3_)O_3_(*x* = 0–1) ceramics by the one-step synthesis method. The effects of Ca^2+^ addition on the phase composition, B-site ion long-range order (LRO) and microwave dielectric properties of BZN ceramics were studied. The obtained results showed that, due to the evaporation of ZnO, the second phases of Ba_5_Nb_4_O_15_ and CaNbO_3_ were formed during the sintering process, and their peak intensities increased with the increase of *x*, which might damage the microwave properties of the samples. The ceramics could form LRO in the case of low BZN content, which was reflected in the existence of a superlattice reflection in the sample (0.1 ≤ *x* ≤ 0.5) at a low angle [47]. With the increase of BZN content, the *Q* × *f* value, dielectric constant and τ*_f_* value of the samples all increased. Ceramics have the best properties with *x* = 0.1: ε_r_ = 24, *Q* × *f* = 23,510 GHz (*f* = 10–15 GHz), τ*_f_* = −9 ppm/°C.

### 4.2. B-Site Ion Doping

A. Mergen and E. Korkmaz [48] prepared BZN ceramics by doping with In, Ce and Bi (molar ratio: between 0.2 mol% and 4.0 mol%) using mixed oxide technology to improve the properties of BZN ceramics. It was found that, except for the sample doped with 0.5 mol% Bi, there was no secondary phase in the Bi-doped samples sintered at 1300 °C and Ce-doped samples sintered at 1400 °C. The above results showed that pure BZN could be acquired at high temperatures. The ion radius of In^3+^ or Ce^4+^ was lager than that of Zn^2+^. In this case, the substitution of In^3+^ and Ce^4+^ for Zn^2+^ in the BZN structure resulted in the increase of the lattice constant. However, Bi^3+^ doping did not increase the lattice constant, which was due to the formation of the secondary phase and the evaporation of ZnO and Bi_2_O_3_. The relative density of In-doped BZN samples sintered at 1300 °C for 4 h increased with the increase of In^3+^, and reached a maximum of 99.79% when the doping content of In^3+^ was 4.0 mol%. The relative density of BZN samples doped with Bi^3+^ was not consistent with the amount of Bi^3+^. The changes of relative density and lattice constant of BZN doped with In^3+^, Ce^4+^, and Bi^3+^ are shown in Table 3 and Table 4, respectively.

C.W. Ahn et al. [18] added Co^2+^ to BZN by conventional solid-state synthesis to adjust the dielectric properties, obtaining (1-*x*)Ba(Co_1/3_Nb_2/3_)O_3_-*x*Ba(Zn_1/3_Nb_2/3_)O_3_ composite ceramics. It was found that when *x* ≤ 0.3, a 1:2 ordered structure formed, and the *Q* × *f* value was larger than 70,000 GHz (*f* = 8.5–9.0 GHz). At this moment, the microstructure of the sample was similar to that of Ba(Co_1/3_Nb_2/3_)O_3_. However, when *x* > 0.3, the 1:2 ordered structure of composite ceramics disappeared, the *Q* × *f* value decreased to 55,000 GHz (*f* = 8.5–9.0 GHz), and the microstructure of the sample was similar to that of BZN. The sintering temperature of 1400 °C was the inflection point for the change of the *Q* × *f* value. When the sintering temperature was less than 1400 °C, the *Q* value was about 60,000 GHz (*f* = 8.5–9.0 GHz). When the sintering temperature was equal to 1400 °C, the *Q* × *f* value reached 78,000 GHz (*f* = 8.5–9.0 GHz). When the sintering temperature exceeded 1400 °C, the *Q* × *f* value decreased suddenly. The above changes were mainly due to the obvious changes in the microstructure of the samples and the formation of a large number of liquid phases [49].

A.F. Qasrawi et al. [50] added Sb^5+^ ions to BZN in order to improve the dielectric properties, obtaining Ba(Zn_1/3_Nb_2/3−*x*_Sb*_x_*)O_3_. It was found that when *x* was greater than 0.4, the Sb^5+^ ion reached the upper limit of solubility. With Sb^5+^-doping, Ba(Zn_1/3_Nb_2/3−*x*_Sb*_x_*)O_3_ was decomposed into hexagonal ZnSb, monoclinic BaSb_2_, and monoclinic BaNb_2_. When the *x* value increased from 0.10 to 0.30, the grain size reduced from 47 nm to 38 nm, which was due to the replacement of the smaller ion Sb^5+^ (0.62 Å) to the larger ion radius Nb^5+^ (0.69 Å) [51]. When *x* = 0.30, the density of Ba(Zn_1/3_Nb_2/3−*x*_Sb*_x_*)O_3_ ceramic system reached the maximum, and the corresponding relative density was 98.91%. When the upper limit of Sb^5+^ ion solubility was exceeded, the relative density of the sample decreased to 94.50%.

J.Z. Li et al. [52] synthesized zinc-rich non-stoichiometric Ba(Zn_1/3_Nb_2/3_)_1−*x*_Zn*_x_*O_3_(*x* = 0.01–0.04) ceramics by a conventional solid-state reaction process. It was found that the main phase of the sample was the BZN phase with a simple cubic perovskite structure, followed by the Ba_5_Nb_4_O_15_ phase. At *x* = 0.02, the ceramic had the smallest unit cell volume. Due to the evaporation of the Zn element, more and more impurities were generated on the surface of the sample to hinder densification, which made the Zn content on the surface of the sample higher than that in the internal grains. Raman results showed that when *x* increased from 0.02 to 0.04, the Ba(Zn_1/3_Nb_2/3_)_1−*x*_Zn*_x_*O_3_ structure changed from a 1:2 to a 1:1 ordered structure. The micrography showed that, for *x* = 0.02, the samples possessed uniform grains, clear outlines, good compactness, and minimal dielectric loss (tanδ = 5.5 × 10^−4^).

**Figure 1:**
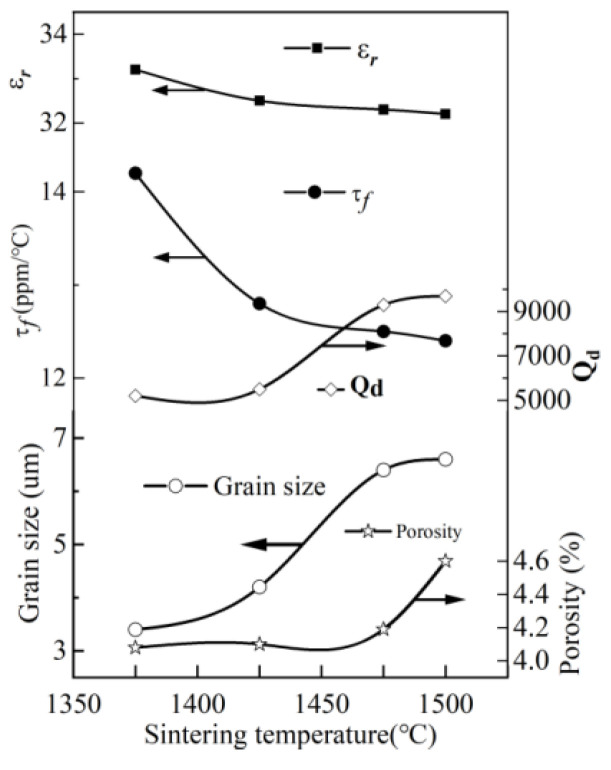
Microwave dielectric properties of 0.774Ba(Zn_1/3_Nb_2/3_)O_3_-0.226BaSnO_3_ sintered at different temperatures for 6 h; data from Ref. [53].

**Figure 2:**
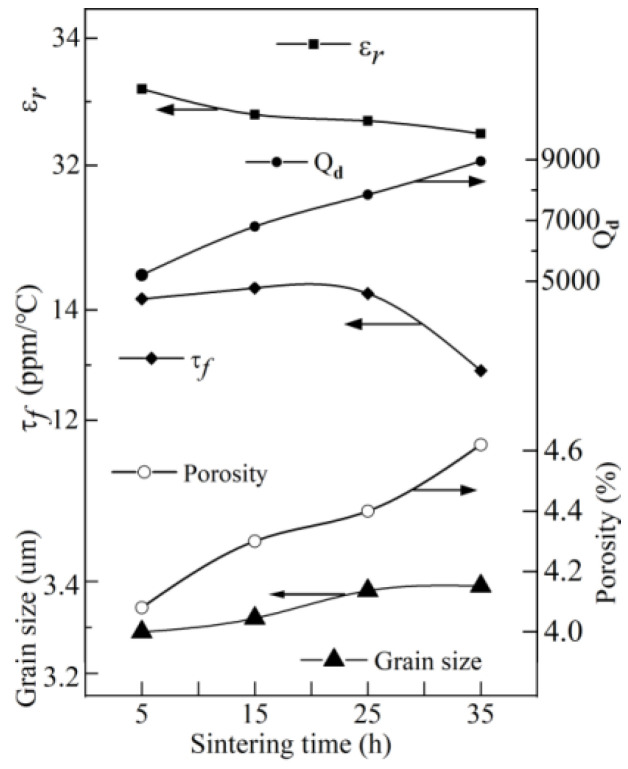
Effect of different sintering time at 1380 °C on microwave dielectric properties of 0.774Ba(Zn_1/3_Nb_2/3_)O_3_-0.226BaSnO_3_; data from Ref. [53].

J.S. Sun et al. [53] added Sn^4+^ ions to BZN in order to improve the dielectric properties, obtaining (1-*x*)Ba(Zn_1/3_Nb_2/3_)O_3_-*x*BaSnO_3_ (BSZN, 0 ≤ *x* ≤ 0.32). The content of Sn^4+^, Zn^2+^, and Nb^5+^ decreased in the sintering process. With the extension of sintering time, the number of pores increased and grain growth was inhibited. With the increase of BaSnO_3_ content, the dielectric constant and τ*_f_* value of ceramic samples decreased. When the content of BaSnO_3_ increased to 2 mol%, the dielectric constant ε_r_ = 40, *Q* = 7500 (*f* = 10 GHz). When the content of BaSnO_3_ was more than 30 mol%, the *Q* value decreased to 4100. When the BSZN composite was sintered at 1500 °C for 6 h, the *Q* value reached 9700. At this moment, the dielectric constant was ε_r_ = 32, τ*_f_* = +12 ppm/°C. Considering the small τ*_f_* value and appropriate *Q* value, the authors selected 0.774Ba(Zn_1/3_Nb_2/3_)O_3_-0.226BaSnO_3_ as the research subject. Figure 1 shows the microwave dielectric properties of 0.774Ba(Zn_1/3_Nb_2/3_)O_3_-0.226BaSnO_3_ sintered at different temperatures for 6 h. It was found that the 0.774Ba (Zn_1/3_Nb_2/3_)O_3_-0.226BaSnO_3_ ceramic system sintered at 1500 °C for 6 h had appropriate dielectric properties: τ*_f_* = +12 ppm/°C and *Q* = 9700 (*f* = 10 GHz). The effects of different sintering temperatures and times on the microwave dielectric properties of 0.774Ba(Zn_1/3_Nb_2/3_)O_3_-0.226BaSnO_3_ are shown in Figure 1 and Figure 2.

### 4.3. Oxide Doping

M.H. Kim et al. [54] added B_2_O_3_ and CuO to BZN to form composite ceramics to improve the dielectric properties of BZN. When the doping amount of B_2_O_3_ exceeded 5.0 mol% and CuO exceeded 20.0 mol%, the secondary phases of BaB_4_O_7_, BaB_2_O_4_ and BaNb_2_O_6_ were obvious, and BaB_4_O_7_, BaB_2_O_4_ existed in the liquid phase during sintering process, which was also conducive to the densification of samples. The permittivity of the sample was correlated with the change of sintering temperature. Specifically, the dielectric constant of the sample sintered at 950 °C with 2.0 mol% B_2_O_3_ was close to that of BZN sintered at 1350 °C. When the doping amount of B_2_O_3_ was 2.0 mol%, the *Q* value of the sample reached a maximum. However, when the doping amount of B_2_O_3_ exceeded 2.0 mol%, the formation of secondary phases of BaB_2_O_4_ and BaNb_2_O_6_ deteriorated the *Q* value. After adding 1.0 mol% B_2_O_3_ and 5.0 mol% CuO to BZN, dense microstructures were obtained for the sample and the average grain size reached 300 nm. During the sintering process, CuO, B_2_O_3_ and Ba^2+^ reacted with each other to form BaB_4_O_7_ and a secondary phase containing Ba^2+^ and Cu^2+^, while the secondary phase containing Ba^2+^ and Cu^2+^ existed in a liquid phase, which was beneficial to the densification of BZN ceramics. Ba(Zn_1/3_Nb_2/3_)O_3_ + 1.0 mol% B_2_O_3_ + 5.0 mol% CuO ceramic system was sintered at 850 °C for 2 h, obtaining good dielectric properties: ε_r_ = 36, *Q* = 19,000 (*f* = 6.5 GHz), and τ*_f_* = 21 ppm/°C.

M.H. Kim et al. [55] first synthesized BaCu(B_2_O_5_) and then added BaCu(B_2_O_5_) to BZN so as to form composite ceramics. BaCu(B_2_O_5_) contributed to the densification of BZN ceramics. With 6.0 mol% BaCu(B_2_O_5_) addition, a dense microstructure was formed. The permittivity, *Q* value, and relative density also reached a maximum when the content of BaCu(B_2_O_5_) was 6.0 mol%. However, if BaCu(B_2_O_5_) content continued to increase, a large number of secondary phases were obvious, resulting in a decline of permittivity. BZN ceramics with 6.0 mol% BaCu(B_2_O_5_) sintered at 875 °C for 2 h possessed excellent dielectric properties: ε_r_ = 35, *Q* = 16,000 (*f* = 8.9 GHz), and τ*_f_* = +22.1 ppm/°C.

### 4.4. Other Research Methods

The impact of stoichiometric composition deviation and heat treatment on the crystal phase and *Q* value in the BZN system was investigated by E. Koga et al. [56]. The results showed that heat treatment and minor compositional variation from stoichiometric BZN could affect structural order and crystallographic phase. The order–disorder phase transition happened around 1300–1400 °C for stoichiometric BZN. Regardless of the crystallographic arrangement, stoichiometric BZN with a greater density possessed a high *Q* value. Non-stoichiometric disordered BZN with secondary phase or low density was created due to a minor variation in composition from stoichiometry. It was reported that secondary phases, lattice flaws, and poor density contributed to lowering the *Q* value.

H. Hughes et al. [57] prepared (1-*x*)Ba(Zn_1/3_Nb_2/3_)O_3_-*x*Ba(Ga_1/2_Ta_1/2_)O_3_ ceramics by the mixed oxide method. The effect of Ba(Ga_1/2_Ta_1/2_)O_3_ (BGT) doping on the microstructure of BZN was studied. It was found that the thickness of the secondary phase was about 25 μm under the conditions of sintering at 1350 °C for 4 h, which might have been formed by the evaporation of Zn. Under the same conditions, the thickness of the secondary phase of the BZN sample doped with 20% BGT reached 50 μm. Continuing to increase the BGT, the secondary phase content increased. Compared to pure BZN, the average grain size of BZN samples doped with 20% BGT increased to 8 μm. A new impurity phase Ba_3_Ga_4_O_9_ was found after the analysis of the samples highly doped with BGT. There were few discussions on this phase and it deserves further study.

Some grain boundary glass phases are often introduced in ceramic media due to the addition of co-solvents or unsuitable sintering and cooling temperatures. The introduction of these glass phases could lower the sintering temperature of the ceramic system and increase the bulk density. However, it could change the main crystal phase, resulting in an increase of dielectric loss. In 2006, F. Roulland et al. [58] studied the effect of glass phase doping on the sintering properties and microwave dielectric properties of BZN ceramics. It was found that the doping of the glassy phase could effectively decrease the sintering temperature of the ceramic system without changing the main properties of the dielectric material. Among them, doping with 5 mol% LiF and 10 mol% B_2_O_3_ could decrease the sintering temperature by 350 °C. In addition, after co-sintering BZN ceramics with silver, the obtained samples showed that no secondary phase was obvious, the dielectric constant was 37.2, and the dielectric loss was also small.

A.G. Belous et al. [59] investigated the effect of A-site or B-site non-stoichiometry on the structure and dielectric properties of Ba(A_1/3_Nb_2/3_)O_3_ (A = Co, Zn) ceramics. It was found that the microstructure, *Q* value and phase composition of the samples were easily affected by deviations in the stoichiometric composition. This deviation very easily form a barium-rich second phase with a hexagonal perovskite structure, similar to Ba_8_A^2+^Nb_6_O_24_, or to a niobium-rich ferroelectric phase with a tetragonal tungsten bronze structure, similar to Ba_6_CoNb_9_O_30_. The creation of these secondary phases was always accompanied by a decrease in the dielectric properties of the ceramic system. The data presented in this paper showed that the Co-deficient BCN (*Q* × *f* = 85,000 GHz) and Ba-deficient BZN (*Q* × *f* = 90,000 GHz) (*f* = 10 GHz) samples obtained good *Q* values after sintering for 8 h without annealing behavior.

## 5. Problems and Solutions in Improving the Performance of BZN Ceramics

### 5.1. Problems in Improving the Performance of BZN Ceramics

Ba(Zn_1/3_Nb_2/3_)O_3_ ceramics are usually prepared by the conventional solid-phase method, which consists of ball milling a certain stoichiometric ratio of raw materials (BaCO_3_, ZnO, Nb_2_O_5_), calcinating in the range of 1100 °C–1200 °C and subsequent high-temperature sintering in the range of 1400 °C–1500 °C. However, the volatilization of ZnO at high temperatures led to the presence of secondary phases, such as Ba_5_Nb_4_O_15_ [60], during sintering, which damaged the dielectric properties of BZN ceramics to some extent.

W.T. Xie et al. [61] prepared (1-*x*)ZnNb_2_O_6_-*x*Ba(Zn_1/3_Nb_2/3_)O_3_ (*x* = 0.25, 0.30, 0.35, 0.40) composite ceramics by a conventional solid-state method to study their properties. It was found that the secondary phase Ba_3_Zn_1/3_Nb_14/3_O_15_ and liquid phase were formed because of the evaporation of ZnO in the sintering process, and the liquid phase assisted grain growth. As the mole fraction of BZN increased, the bulk density, dielectric constant and *Q* value of the samples first increased and then decreased, while, the τ*_f_* value increased directly from −12.68 to +10.34 ppm/°C. It was believed that the above phenomenon was reasonable. As the dielectric properties of BZN are better than those of ZnNb_2_O_6_, the appropriate amount of BZN could improve the order degree of ceramics, thereby promoting the densification sintering of ceramics. However, surplus BZN would cause excessive evaporation of the Zn element, resulting in the formation of redundant secondary phases.

S.Y. Noh et al. [17] used conventional solid-state synthesis to prepare BZN and thereby researched the effects of structural changes on the microwave dielectric properties of BZN ceramics. It was found that the evaporation of the Zn element had an important influence on the structure and properties of the ceramic system during the sintering process. For the samples sintered at above 1350 °C, some liquid phases were observed, due to the evaporation of ZnO, and the presence of the liquid phases promoted grain growth. At the same time, the loss of Zn ions also deteriorated the 1:2 ordering structure of the ceramics. The secondary phases of Ba_5_Nb_4_O_15_ and BaNb_6_O_16_ decreased the relative density and *Q* value of the samples, caused by the evaporation of ZnO.

### 5.2. Solutions in Improving the Performance of BZN Ceramics

For microwave dielectric ceramics, there are many factors resulting in dielectric loss. In general, dielectric loss includes intrinsic loss and extrinsic loss. Intrinsic loss refers to crystal loss due to the anharmonic interaction between the electromagnetic field and lattice phonon system. Intrinsic losses are very sensitive to crystal symmetry, and crystals with different symmetries exhibit different frequency and temperature dependencies. Extrinsic losses originate from defects in the material structure and microstructure, such as pores, grain boundaries, dislocations, stacking faults, ion vacancies, etc. [62]. In many cases, extrinsic loss i larger than intrinsic loss. The quality factor would then be dominated by the extrinsic loss, and the optimization of the quality factor required reducing the extrinsic loss. Some factors affecting extrinsic loss are as follows:

(1) Raw materials and preparation conditions

Raw materials play a key role in ensuring the quality of microwave dielectric ceramics. The purity of raw materials directly affects the quality factor. Zhang found in the study of Zr*_x_*Ti_1−*x*_O_4_ (*x* = 0.4–0.6) ceramics that Fe impurities led to a decrease in *Q* values [63]. The grain size and distribution of raw materials also affect the sintering process and, thus, the final *Q* value. Shi [64] compared the *Q* × *f* value of MgTiO_3_ ceramics prepared from fine powder and commercial powder raw materials, and found that the *Q* × *f* value of the former (368,000 GHz) was much higher than that of the latter (160,000 GHz), which was attributed to the uneven grain distribution caused by the inhomogeneous raw powder. The difference in preparation conditions leads to a huge difference in *Q* × *f* value, which includes pre-sintering conditions and sintering conditions (heating rate, sintering temperature and time, sintering atmosphere, cooling rate, etc.). Lee [65] found that double pre-firing could promote the growth of Ba(Zn_1/3_Nb_2/3_)O_3_ grains and improve the uniformity of ceramics, compared to single pre-firing, thus greatly improving the *Q* × *f* value. For composite perovskites, the *Q* × *f* value is particularly sensitive to sintering conditions, and the longer the stay time at the order–disorder phase transition temperature, the more conducive to the ordering of ions, thus improving the *Q* × *f* value [66,67]. For microwave dielectric ceramics with volatile elements, the volatilization of the elements is an important factor affecting the microwave dielectric properties. In this regard, the sintering method of buried firing was usually used to suppress the volatilization of components and improve the *Q* × *f* value [67].

(2) Non-stoichiometric ratio

The optimal *Q* × *f* values of microwave dielectric ceramics are not all obtained under strict stoichiometric ratios. It is known that higher *Q* × *f* values can be obtained by changing the stoichiometric ratio of certain elements. The improvement of *Q* × *f* value is usually obtained when the element content is lower than the stoichiometric value, the reason being that ion vacancies caused by element deficiency are beneficial to mass transfer and ion ordering. For example, Durilin [68] found that when Ba or Zn was deficient, the sintering performance of Ba(Zn_1/2_W_1/2_)O_3_ improved, and the 1:1 order degree of B site improved, resulting in a higher *Q* × *f* value. Wu and Davies [16] unexpectedly found that an excess of Nb increased the *Q* × *f* value of Ba(Zn_1/3_Nb_2/3_)O_3_ and the excess of Nb also led to the creation of Ba or O vacancies, thus improving the ordering degree. However, not all non-stoichiometric compounds can improve the *Q* × *f* value. As for CoNb_2_O_6_ reported by Belous [69], the deficiency or excess of Co would lead to a decrease in *Q* × *f* value due to the formation of the secondary phase.

(3) Secondary phase and microstructure

The phase composition in microwave dielectric ceramics has a close relationship with the *Q* × *f* value. Regarding Ba([Mg,Zn]_1/3_Ta_2/3_)O_3_ Ichinose and Shimada [70] reported that the secondary phase BaTaO_6_ was generated due to the volatilization of Zn, which reduced the *Q* × *f* value. The grain structure of microwave dielectric ceramics also affected the dielectric loss. Ceramics are generally composed of a large number of crystal grains, and the grain boundary between crystal grains is a structural defect, so dielectric loss occurs. Generally speaking, the grain boundary density of larger grains lowers, resulting in less dielectric loss [71,72]. This does not mean that the larger the grains, the smaller the dielectric loss, because further growth of the grains produces another dielectric loss factor. For example, during the cooling process, when the grain grows further, it is not easy for oxygen to enter the interior of the grain so as to eliminate the oxygen vacancies in the lattice, so that the content of oxygen vacancies in the ceramic increase, resulting in greater dielectric loss [73]. When the sintering temperature is too high, liquid phase sintering occurs, the crystal grains grow abnormally and form closed pores, which also increases the dielectric loss [74]. The abnormal growth of grains often produces a lot of point defects, line defects, surface defects, body defects and other defects in the crystal structure [75]. These all contribute to the dielectric loss.

(4) Cation ordering degree

Another research focus of the microwave ceramic system is the order/disorder of B-site ions in composite perovskite ceramics. The ordering degree depends on the ion radius difference and charge difference, which is a very important factor affecting the *Q* × *f* value. Generally speaking, the *Q* × *f* value increases with the increase of the cation ordering degree.

Ba(Zn_1/3_Nb_2/3_)O_3_ ceramics are generally sintered at high temperatures of above 1400 °C, whereas their ordered–disordered phase transition temperature is about 1375 °C. Therefore, the ordered structures are transformed into disordered structures during the high temperature sintering process, resulting in a decrease of the *Q* × *f* value [76]. In addition, Zn elements tend to be volatilized during the high temperature sintering process, which damages the order degree and introduces the impurity phase, which further reduces the *Q* × *f* value, and is not conducive to obtaining high-performance Ba(Zn_1/3_Nb_2/3_)O_3_ microwave dielectric ceramics. Thus, it can be seen that reducing the sintering temperature of Ba(Zn_1/3_Nb_2/3_)O_3_, or inhibiting the volatilization of ZnO, benefits and improves the dielectric properties of Ba(Zn_1/3_Nb_2/3_)O_3_ ceramics.

(5) ion valence state

As for the variation of valence elements in the material, the valence state of the element and its change affects many physical properties of the material. For microwave dielectric ceramics, dielectric loss, as a physical property, should also be affected by the valence state of elements [77]. Templeton [77] found that in TiO_2_ ceramics, Ti^4+^ would be reduced to Ti^3+^ to generate oxygen vacancies and increase dielectric loss. Ti^3+^ might be re-oxidized when the temperature lowered. However, as the density of TiO_2_ ceramics increased, it was difficult for O_2_ to enter the ceramic body to re-oxidize Ti^3+^, resulting in increased loss. In the composite perovskite system, Endo [23] found that the *Q* × *f* value of Ba(Co_1/3_Nb_2/3_)O_3_-Ba(Zn_1/3_Nb_2/3_)O_3_ in N_2_ atmosphere was higher than that in O_2_ atmosphere and air, suggesting that the difference in *Q* × *f* values obtained under different atmospheres might be related to the valence state of Co.

## 6. Application of BZN Ceramics

The dielectric resonator made from microwave ceramics can be considered a dielectric waveguide, or an open coaxial resonant cavity, and microwaves with a specific frequency are enclosed in a dielectric space with an open circuit at both ends. Such devices are available in a variety of shapes and configurations, which are commonly made into cylindrical, coaxial or ribbon line, rectangular and round rod shapes according to patterns, such as TE_01δ_ and TEM. Microwave networks can not only n be coupled with capacitive set parameters or distributed parameters to form filters, but can also be coupled with microwave transistors to form oscillators and microwave devices, such as MIC and MMIC. Moreover, they are widely used in radar, navigation, microwave communication, radio transmitting base stations and other facilities [78,79,80,81].

A dielectric resonator is a typical application for microwave dielectric waveguides. The dielectric waveguide can be made into circular rod-shaped or square rod-shaped transmission lines, including dielectric resonators, rod antennas, etc. [82,83,84]. Microwave dielectric substrates are another form of microwave ceramic dielectric application [85,86], mainly used in microwave and millimeter wave satellite communications, military radar, electronic navigation, microwave security monitoring, etc.

Dielectric filters usually consist of several resonators connected longitudinally in multiple stages. They have some distinctive features, such as extremely small differential losses and excellent power resistance. In real-life, the applications of dielectric filters are wide and universal, including mobile phones, car phones, bandpass and band stop filters of base stations, integrated receivers and transmitter duplexers. TEM mode coaxial dielectric resonator filters, antenna duplexers, and dielectric filters, consisting of multiple coaxial resonators, are also in possible [87,88,89,90,91,92,93].

H.M. Sung proposed the idea of a low temperature co-fired multilayer dielectric planar type filter [94]. It was successfully implemented in Japan by SMI, Matsushita-Nitto, Soshin, NGK, Philips, JTI, etc. From 1984 to 1989, Murata Corporation broke through the technical problems in the printed copper conductor coil and low temperature co-firing dielectric material system, and overcame the dispersion of L and C, obtaining a stable resonant frequency, so that the chip multilayer LC filter could be widely used in the high frequency microwave band. TDK developed a low temperature co-firing medium material system with a silver inner electrode sintered below 960 °C. It was used to successfully develop DEA series chip multi-layer LC filters. In 1999, the improved DLA16 created a new record for miniaturization of 1608 size. Multilayer filters are gradually being promoted in wireless phones, such as DECT, and PHS, and in cellular phones, such as PDC, PCN, PCS, GSM and W-CDMA, which are suitable for surface mounting. In recent years, it has tended to be used in the 2.4–5.8 GHz band for systems, such as W-LAN [95].

In short, microwave dielectric ceramics with excellent dielectric properties possess a series of circuit functions, such as dielectric isolation, dielectric waveguide, dielectric resonance, etc., and are widely used in satellite communications (SC, 20–30GHZ), satellite live TV (SLDTV, 2–13 GHZ), electronic countermeasures, navigation [96], radar, cellular mobile communications (0.4–1 GHZ), computers, etc.

## 7. Summary and Outlook

In this article, recent advances in BZN ceramics were summarized, including different ceramic preparation methods and performance modification of BZN ceramics by the substitution of Ba(Zn_1/3_Nb_2/3_)O_3_ with new substances, changing the stoichiometric ratio of BZN raw materials, adding firing aids and other measures. Superior dielectric properties are obtained with an adjustable dielectric constant, a high-quality factor, and near zero temperature coefficient of resonance frequency.

However, there are still some problems for Ba(Zn_1/3_Nb_2/3_)O_3_ microwave dielectric ceramics. Ba(Zn_1/3_Nb_2/3_)O_3_ ceramics are sintered at a very high temperature of above 1400 °C. The addition of sintering additives generally deteriorates the dielectric properties to some extent, making it difficult to regulate the relationship between low temperature and excellent performance. The research of microwave dielectric ceramics has also focused on ion replacement, synthesis processes, and related research. It is common for most studies to be empirical, relying heavily on experiments and very little on theoretical analyses through simulations and computations. This is not yet sufficient to conduct relevant theoretical research. In particular, the mechanism of crystal structure change, the dielectric loss mechanism and microwave performance still need to be studied in detail.

**Figure 3:**
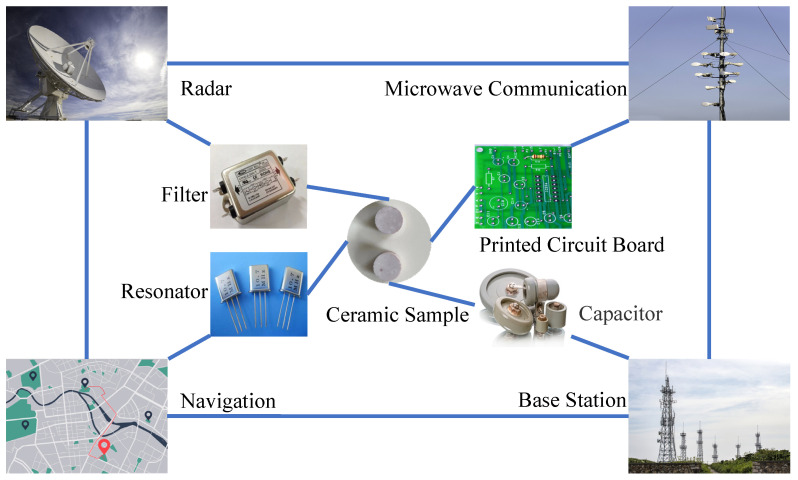
Main applications of microwave dielectric ceramics.

Since 5G communication technology has been commercialized, microwave technology has gradually progressed towards miniaturization, multi-frequency and high integration. Especially in civilian use (handheld mobile devices), rapid development in a large output and low-cost direction (as shown in Figure 3) has enabling the development of microwave dielectric ceramics to be a research hot spot. In view of current and future technical needs, research on BZN series ceramics should focus on several aspects. First, further improvement in the microwave dielectric properties of ceramic material and continued development of new BZN ceramic systems. Second, the advanced test characterization techniques (transmission electron microscopy, Raman spectroscopy, X-ray photoelectron spectroscopy, etc.) required to investigate the dielectric response mechanism of chalcogenide ceramics from the crystal structure perspective. In addition, complex chemical bonding calculations are necessary to explore the connection between intrinsic factors and microwave properties. Finally, it is necessary to study the phase solubility between BZN ceramics and sintering additives, and explore the cooling mechanism of sintering additives in order to develop new sintering additives.

## Figures and Tables

**Table 1:** The microwave dielectric properties of A(X_l/3_Y_2/3_)O_3_ ceramic compounds.

Ceramic Composition	Fabrication Method	ε_r_	*Q* × *f* (GHz)	τ*_f_* (ppm/°C)	Reference
Ba(Zn_1/3_Nb_2/3_)O_3_	Solid-state ceramic route	41	86,925	+31	[19]
Ba(Zn_1/3_Ta_2/3_)O_3_	Solid-state ceramic route	28	168,000	+0.5	[7]
Ba(Mg_1/3_Nb_2/3_)O_3_	Solid-state ceramic route	32	55,440	+33	[19]
Ba(Mg_1/3_Ta_2/3_)O_3_	Mixed oxide method	25	250,000	+2	[20]
Ba(Co_1/3_Nb_2/3_)O_3_	Mixed oxide method	32	66,500	−10	[21]
Ba(Co_1/3_Ta_2/3_)O_3_	Mixed oxide method	32.6	86,857	−14	[22]
Ba(Ni_1/3_Nb_2/3_)O_3_	Solid-state ceramic route	31.2	38,000	−0.2	[23]
Ba(Ni_1/3_Ta_2/3_)O_3_	Solid-state ceramic route	23	49,700	−18	[24]

**Table 2:** Microwave dielectric properties and cell volumes for (Ca_1−*x*_Ba*_x_*)(Zn_1/3_Nb_2/3_)O_3_ ceramics (*f* = 5.4–6.7 GHz); data from Ref. [41].

*x*	ε_r_	*Q* × *f* (GHz)	τ_*f*_ (ppm/°C)	Cell Volume (Å^3^)		
				Cubic	Hexagonal	Orthogonal
0	34	11,840	−35			240.5637
0.1	36	16,170	−12		209.3487	240.6796
0.3	41	3470	62		207.7826	240.2788
0.5	47	1920	128		207.3537	240.3285
0.7	54	1480	183		207.3010	237.0761
0.9	53	300	217		205.9120	
1.0	40	29,710	23	68.8234		

**Table 3:** Relative density of doped BZN samples sintered at different temperatures. Reprinted with permission from [48], copyright 2011, Elsevier.

	Relative Density (%)					
	0.2 mol%	0.5 mol%	1.0 mol%	2.0 mol%	4.0 mol%	Sintering Temperature
In	91.86	97.59	97.99	99.67	99.79	1300 °C
Ce	97.85	95.25	96.71	96.95	92.84	1400 °C
Bi	94.68	91.44	90.70	95.92	95.87	1250 °C

Theoretical density of undoped BZN ceramics is 6.516 g/cm^3^.

**Table 4:** Lattice parameters of doped BZN samples. Reprinted with permission from [48], copyright 2011, Elsevier.

	Lattice Parameter (Å)					
	0.2 mol%	0.5 mol%	1.0 mol%	2.0 mol%	4.0 mol%	Sintering Temperature
In	4.0941	4.0962	4.1008	4.1155	4.1202	1300 °C
Ce	4.1369	4.1379	4.1434	4.1437	4.1543	1400 °C
Bi	4.0913	4.0893	4.0862	4.0922	4.0935	1250 °C

Lattice parameters of undoped BZN samples ceramic is 4.09408 Å.

## Data Availability

Not applicable.
